# Lobodontia‐Affected Teeth Exhibit Compromised Integrity and Enamel Defects: A Deep Phenotyping Study

**DOI:** 10.1111/odi.15390

**Published:** 2025-05-25

**Authors:** Thanakorn Theerapanon, Narin Intarak, Sasiprapa Prommanee, Sunisa Somkana, Sirinya Kulvitit, Anucharte Srijunbarl, Junji Tagami, Thantrira Porntaveetus

**Affiliations:** ^1^ Interdisciplinary Program of Biomedical Sciences, Graduate School Chulalongkorn University Bangkok Thailand; ^2^ Center of Excellence in Precision Medicine and Digital Health, Center of Excellence in Genomics and Precision Dentistry, Geriatric Dentistry and Special Patients Care International Program, Department of Physiology, Faculty of Dentistry Chulalongkorn University Bangkok Thailand; ^3^ Clinical Research Center, Faculty of Dentistry Chulalongkorn University Bangkok Thailand; ^4^ Department of Operative Dentistry, Faculty of Dentistry Chulalongkorn University Bangkok Thailand; ^5^ Dental Materials R&D Center, Faculty of Dentistry Chulalongkorn University Bangkok Thailand; ^6^ Faculty of Dentistry Chulalongkorn University Bangkok Thailand; ^7^ Department of Cariology and Operative Dentistry, Graduate School of Medical and Dental Sciences Institute of Science Tokyo Tokyo Japan; ^8^ Clinic of General‐, Special Care and Geriatric Dentistry, Center for Dental Medicine University of Zurich Zurich Switzerland

**Keywords:** dens invaginatus, health disparity, healthcare, multitubercular molar, pyramidal root, taurodontism

## Abstract

**Objective:**

Lobodontia, a complex tooth disorder characterized by carnivore‐like dentition, presents unique clinical challenges. This study aimed to characterize the physical, mechanical, and ultrastructural properties of lobodontia teeth to inform precision treatment strategies.

**Methods:**

This study included two extracted teeth (one from each lobodontia patient) and ten control teeth from healthy individuals. Clinical, radiographic, dental impression, and microstructural analyses were performed, including colorimetry, surface roughness, microCT, SEM, EDX, nanoindentation, and histology.

**Results:**

Lobodontia teeth exhibited a distinctive multitubercular occlusal morphology, with deep grooves and multiple cusps. Radiographs showed dens invaginatus and taurodontism. Compared to controls, lobodontia teeth had increased surface roughness and color deviations. Although mineral composition was similar, lobodontia teeth showed significantly reduced hardness, elastic modulus, enamel thickness, and a disrupted dentinoenamel junction with a pronounced gap between enamel and dentin.

**Conclusions:**

Lobodontia teeth exhibit compromised structural integrity, including diminished hardness, elasticity, and enamel thickness, alongside a defective dentinoenamel junction. These characteristics increase the risk of biofilm retention, infection, and malocclusion, necessitating personalized care approaches.

## 1 Introduction

Lobodontia, an autosomal dominant condition, is characterized by a spectrum of dental anomalies resembling those seen in carnivores. Individuals with lobodontia exhibit a complex array of dental abnormalities, including multitubercular molar crowns, shovel‐shaped permanent incisors, cone‐shaped premolars and canines, and single pyramidal unbifurcated posterior root forms with a single root canal (Gheorghe et al. 2023; Skrinjaric et al. 2016). Additionally, dens invaginatus, an invagination of enamel within the crown, reduction in tooth size, and malocclusion are commonly observed. The condition has been reported in both primary and permanent dentitions (Gheorghe et al. 2023; Nguyen et al. 1996; Skrinjaric et al. 2016) and affects fewer than 1 in 300,000 individuals (Skrinjaric et al. 2016).

Lobodontia poses significant challenges for dental management due to its profound impact on tooth morphology and structure. The condition manifests as a spectrum of dental anomalies, including shovel‐ or barrel‐shaped incisors, premolars with exaggerated cusps, and dens invaginatus, all of which complicate conventional dental procedures. These aberrant morphologies predispose affected individuals to a higher risk of dental trauma and accelerated wear, compromising oral health. The increased surface area and presence of deep pits and fissures associated with multituberculated crowns create favorable niches for biofilm retention, thereby elevating the risk of caries and infection. Reports of extensive tooth deterioration and premature tooth loss in lobodontia patients underscore the substantial burden this condition places on oral health, highlighting the need for a thorough understanding and finding specialized dental care strategies (Gheorghe et al. 2023).

Lobodontia has been linked to a missense variant (NM_000069.2: c.865A>G, rs139920212) in the *CACNA1S* gene (OMIM*114208), which encodes the Cav1.2 calcium channel (OMIM*114208) (Laugel‐Haushalter et al. 2018). This variant was identified through exome sequencing and exhibits a low allele frequency (< 0.01) in population databases such as gnomAD, TOPMed, and GenomeAsia. The variant co‐segregated with the lobodontia phenotype in an autosomal dominant manner, showing complete penetrance and absence in unaffected individuals (Kantaputra et al. 2024, 2023). Further evidence supporting the role of *CACNA1S* in disease pathogenesis included findings from mutant CACNA1S overexpression in cell culture, which resulted in abnormal cell migration, cytoskeletal alterations, and changes in focal adhesion markers (Kantaputra et al. 2024). Additionally, *Cacna1s* has been shown to be expressed in the dental epithelium during early tooth development (Kantaputra et al. 2023).

While the distinct morphologic features of lobodontia have been documented, the impact of this condition on the mechanical properties and ultrastructure of affected teeth remains unexplored. This study aimed to address this knowledge gap by conducting a comprehensive assessment of lobodontia‐affected teeth, encompassing mineral content, enamel thickness, mineral density, hardness, elasticity, and ultrastructural characteristics. This detailed characterization provides valuable insights into the structural integrity and potential vulnerabilities of lobodontia teeth, informing targeted dental management strategies.

## 2 Materials and Methods

### 2.1 Tooth Samples

The study was approved by the Research Ethics Committee of the Faculty of Dentistry of the University (No. 137/2023, HREC‐DCU2023‐117, Date of approval: 5th January 2024) and in accordance with the Declaration of Helsinki (version 2002) and additional amendments. Informed consent was obtained from all participants.

Permanent mandibular third molars were obtained from two unrelated lobodontia patients (LBD1 and LBD2) and at least three healthy control individuals. Extractions were performed in accordance with the patients' dental treatment plans. Participants with lobodontia and control subjects were free of systemic conditions or medications that could potentially influence dental or bone health. All tooth samples were preserved in 10% formalin solution and stored at 4°C until further analysis. Each LBD tooth was analyzed and compared with control teeth.

### 2.2 Mesiodistal Width

Dental impressions of LBD1 and LBD2 were obtained at ages 23 and 36 years, respectively, to create dental models. The mesiodistal width of each tooth was measured as the greatest distance between the mesial and distal contact points, parallel to the buccal tooth surface, using sliding calipers (TRICLE, Shanghai, China) with an accuracy of 0.05 as previously described (Moorrees and Reed 1964) (Figure S1). Three observers independently performed two measurements each to ensure reliability. *Z*‐scores were calculated for all teeth using reference data from 67 healthy Thai males aged 16 to 32 years (Ruengdit et al. 2011), with a *Z*‐score exceeding ±1.96 indicating a significant deviation from the reference mean.

### 2.3 Tooth Color

A digital intraoral colorimeter (VITA Easyshade V, H. Rauter GmbH & Co., Germany) was used to measure the color of both the buccal and lingual surfaces of the tooth crowns. Color values were expressed using the L*, a*, and b* color scale. This system measures L* for lightness (higher values indicating lighter colors), a* for the red‐green axis (positive values for red, negative values for green), and b* for the yellow‐blue axis (positive values for yellow, negative values for blue). The color difference (∆E value) between Lobodontia (LBD) and control teeth was calculated using an equation described in previous studies (Intarak et al. 2021; Sriwattanapong et al. 2024).

### 2.4 Surface Roughness

Surface roughness was assessed on the buccal and lingual surfaces of the tooth crowns using a surface profilometer (Talyscan 150, Taylor Hobson Ltd., UK). Each surface was measured 30 times randomly at 60‐μm intervals along the *Y*‐axis. The stylus speed was set at 1000 μm per second. The tracing area was 2 mm × 2 mm. A cut‐off length of 0.025 mm was applied. The TalyMap Universal program was used to calculate surface topography parameters.

### 2.5 Mineral Density

The sample was scanned using a micro‐computerized tomographic system (μCT 35, SCANCO Medical, Switzerland). A square area of 20 × 20 pixels (W × H) from 30 layers of sections was selected from each sample. Mineral density was quantified using the Image Processing Language (IPL, Scanco Medical AG). Three spots in the enamel and three spots in the dentine of the patient's teeth were selected to evaluate and compare with the same areas in the controls via cross sections of μCT.

### 2.6 Enamel Thickness

Three sectional μCT images were generated for each sample. The first cross‐sectional image was created in the middle of the scanned teeth. Two additional sections were taken, 30 layers above and below the original section. The tooth crown area (C), the dentine under the enamel cap area (P), and the length of the enamel‐dentine junction (e) were quantified using ImageJ 1.54 software according to a previous study (Smith et al. 2006). The enamel cap area values (c) were derived by subtracting the dentine under the enamel cap area (P) from the tooth crown area (C). The average enamel thickness (AET) was calculated as the ratio of the enamel cap area to the length of the enamel‐dentine junction, represented by the formula AET = c/e.

### 2.7 Tooth Sectioning

Each tooth sample was marked in the middle portion of the tooth and sectioned in a bucco‐lingual plane parallel to its long axis using a slow‐speed precision saw (Isomet 1000 Precision Saw, Buehler) equipped with a diamond disc. The sectioning process was conducted at 450 rpm and under constant water cooling. Four slices with a thickness of 0.5 mm were produced. Subsequently, the obtained tooth slices were polished under water cooling using 1200 grit silicon‐carbide paper and alumina powder. The tooth slices were distributed as follows: one slice for histological examination, one slice for microhardness assessment, one slice for scanning electron microscope (SEM) analysis, and one slice for energy‐dispersive x‐ray (EDX) examination. These were applied to both LBD and control tooth samples.

### 2.8 Nanohardness and Elastic Modulus

Polished tooth sections with a thickness of 0.5 mm were indented using the nanohardness tester (Hysitron TS 77 Select Nanoindenter, Bruker Corporation, US) and a calibrated diamond Berkovich indenter. Indentation was created with a maximum force of 200 mN for 30 spots in the enamel and dentine areas. In all load‐unloading cycles, 50 points were plotted to create the load‐displacement curve. Bruker's Hysitron TriboScan software was used to calculate hardness and elastic modulus values.

### 2.9 Mineral Composition

The dehydrated tooth sections were coated with gold powder for 10 s. Mineral contents, carbon (C), oxygen (O), phosphorus (P), and calcium (Ca) were measured using Energy‐Dispersive X‐ray (EDX) (ISIS 300 EDX‐system; Oxford Instruments, UK) at a magnification of ×1000. Multiple areas of the enamel and dentine were analyzed following a previously established method (Tantibhaedhyangkul et al. 2023). EDX analysis was performed on the occlusal third, middle third, and cervical third of the enamel and dentine within the tooth crown, following the same protocol for both patient and control samples.

### 2.10 Ultrastructure

The tooth sections were etched with 37.5% orthophosphoric acid, rinsed with deionized water, and fixed overnight with 2.5% glutaraldehyde. The samples were dehydrated in a graded ethanol series. After processing critical point drying for 24 h, sections were gold sputter‐coated with gold and scanned using Scanning Electron Microscopy (SEM) (QuantaFeg 250, FEI Company, Oregon, USA). SEM was conducted on the occlusal third, middle third, and cervical third of the enamel and dentine within the tooth crown, following the same protocol for both patient and control samples.

For histological examination, tooth sections were subjected to EDTA decalcification, embedded in paraffin wax, sectioned at a thickness of 5 μm, and stained with hematoxylin and eosin and Masson's Trichrome. The stained sections were examined using a light microscope (DM2000 Microscope with LAS v4.12 program, Leica, Wetzlar, Germany).

### 2.11 Statistical Analysis

Statistical analysis was performed using GraphPad Prism 8 Software (GraphPad Software Inc., CA, USA). Data exhibiting normal distribution were analyzed using independent *t*‐tests. For data with non‐normal distribution, the Mann–Whitney *U* test was employed. Statistical significance was set at *p* ≤ 0.05. A post hoc power analysis was conducted using G*Power version 3.1.9.7 (*f*‐test, ANOVA) with an alpha level of 0.05 to evaluate the adequacy of the sample size in detecting significant group differences (Faul et al. 2009).

## 3 Results

### 3.1 Clinical Dental Anomalies of Lobodontia‐Affected Patients

**FIGURE 1:**
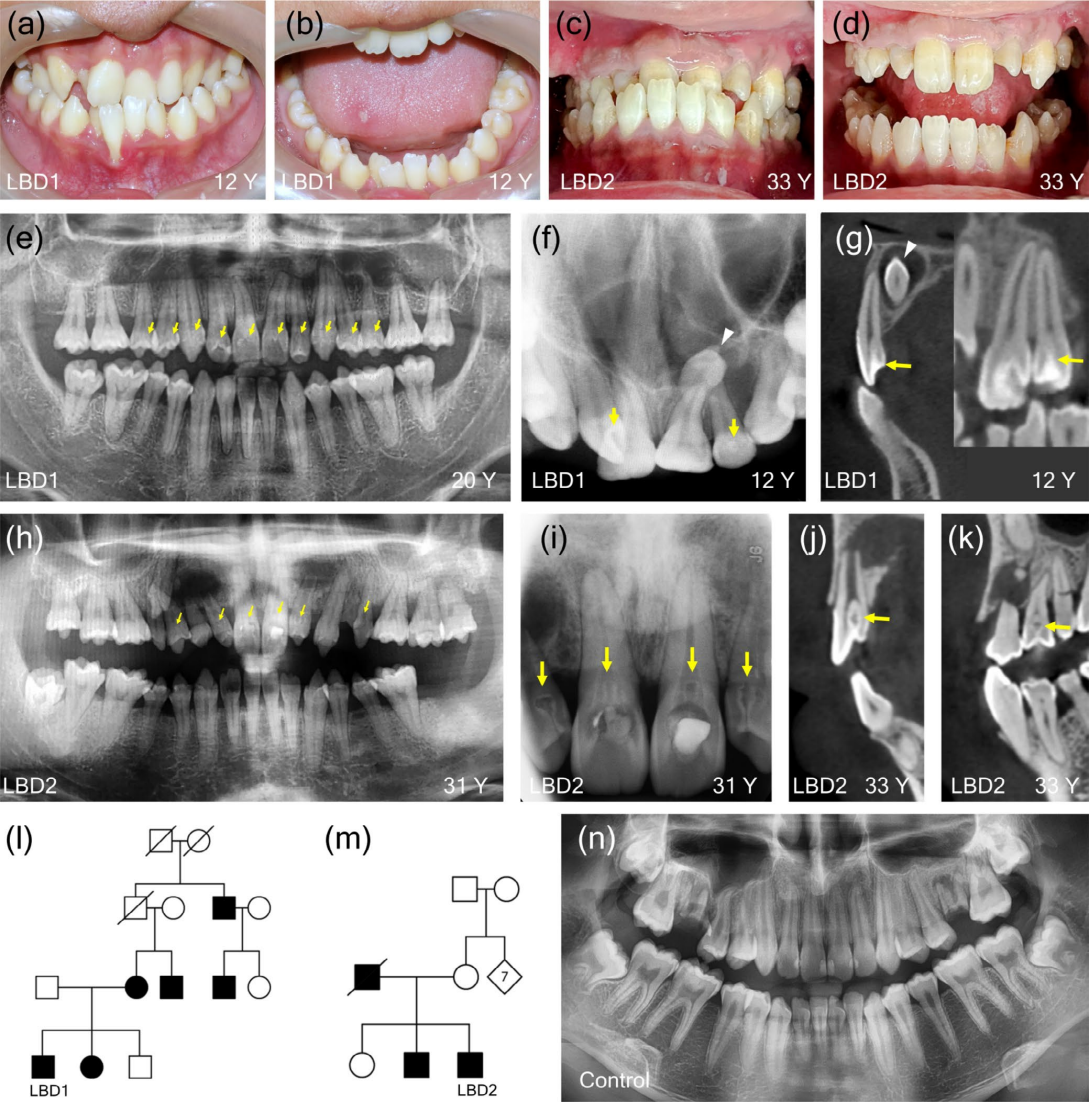
Dental phenotype. Malocclusion and characteristic features of lobodontia, including tritubercular cusps on incisors, single‐pointed cusps on premolars, and multitubercular cusps on molars, were observed in the lobodontia patients 1 (LBD1) (a, b) and the lobodontia patients 2 (LBD2) (c, d). A panoramic radiograph of LBD1 at 20 years of age revealed molars with single pyramidal roots and canals, as well as agenesis of the mandibular left lateral incisor (e). Radiograph (f) and cone‐beam computed tomography (CBCT) (g) of LBD1 at 12 years of age showed mesiodens (white arrow heads) and dens invaginatus in the maxillary incisors, canines, and premolars. Dental radiographs of LBD2 at 31 years of age demonstrated dens invaginatus in the maxillary incisors and premolars, molars with single pyramidal roots and canals, an impacted maxillary right canine with a retained deciduous canine, and a microdont maxillary left second premolar (h, i). CBCT examinations revealed dens invaginatus type II with deep and large invaginations in the maxillary incisors and premolars of LBD2 (j, k). The family pedigrees of the LBD1 (l) and LBD2 (m) indicate an autosomal dominant inheritance pattern. An orthopantomogram of an unaffected control displayed normal crown morphology and typical molar roots with two to three branches (n). Yellow arrows indicate dens invaginatus.

The LBD1 patient, a 12‐year‐old male from Roi Et Province, Thailand, presented with concerns regarding overlapping and misshapen teeth. He presented with multitubercular molars and malocclusion. Thorough oral examinations revealed incisors with three sharp, fang‐like cusps, premolars exhibiting a single pointed cusp, and molars displaying multitubercular morphology (Figure 1a,b). Radiographic and dental cone‐beam computed tomography (CBCT) revealed the presence of inverted mesiodens and dens invaginatus type I in the maxillary incisors, canines, and premolars at the age of 12 years (Figure 1f,g). Additionally, the molars exhibited a single pyramidal root with one canal (Figure 1e).

The patient had caries in the maxillary right central and lateral incisors and mandibular first molars, which were treated with restorations. Fissure sealants were placed on the mandibular left premolars and second molar. Orthodontic treatment was undertaken to address dental crowding, which included the extraction of the third molars, the mandibular left lateral incisor, and a mesiodens. Treatment was successful, achieving stable occlusion and well‐aligned dentition. Post‐treatment follow‐up revealed no complications. The patient is currently wearing a retainer, and the existing restorations remain intact and in good condition. Additionally, oral hygiene has improved following orthodontic treatment. In the family, the patient's younger sister, mother, uncle, and two other relatives also exhibit lobodontia features, indicating the segregation of autosomal dominant lobodontia (Figure 1l).

Dental records for LBD1's mother and younger sister confirmed characteristic features of lobodontia in both individuals, including multitubercular molars with single roots, single‐cusped premolars, and pointed canines. LBD1's younger sister underwent endodontic treatment on her permanent maxillary left lateral incisor at age 10 and has maintained regular dental check‐ups, with a currently stable oral condition. Oral photographs and a panoramic radiograph of LBD1's mother (taken at ages 33 and 41, respectively) revealed typical lobodontia features. She also presented with attrition of the anterior teeth and malocclusion. Additionally, records indicated previous extractions of the maxillary right first molar (due to unrestorable caries) and the maxillary left second premolar (due to malposition and caries) (Figure S2).

The LBD2 patient, a 31‐year‐old male from Nakhon Ratchasima province, Thailand, sought treatment for his maxillary central incisors due to the discoloration. His clinical presentation displayed the characteristic features of lobodontia (Figure 1c,d). Radiographic and CBCT examination revealed dens invaginatus type II with deep and large invaginations in the maxillary incisors and premolars, while the molars presented a single pyramidal shape with one canal. The maxillary right canine was ectopically impacted, and the deciduous maxillary right canine was retained. The maxillary left second premolar was microdont.

The patient presented with pulp necrosis in both maxillary central and lateral incisors, necessitating endodontic treatment (Figure 1h–k). The mandibular left second and third molars were absent at the initial presentation. The retained root of the maxillary left first premolar, deemed unrestorable, along with the maxillary left second premolar exhibiting a root fracture, were both extracted. The maxillary left third molar, diagnosed with generalized stage III, grade C periodontitis, and the vertically impacted mandibular right third molar were also extracted. The patient underwent regular check‐ups, scaling, and root planing, and the oral condition is currently stable. His father and younger brother also exhibited lobodontia, indicating an autosomal dominant inheritance pattern (Figure 1m).

Additionally, both patients exhibited malocclusion, anterior crossbite, and generalized alveolar bone loss. Clinically, normal teeth exhibit a natural shape with well‐defined cusps. Radiographically, they display properly formed tooth crowns with normal cusp morphology, and molars typically have two to three roots (Figure 1n). The LBD1 patient had a history of urinary tract infection at the age of six; however, both patients are currently healthy and report no ongoing medical conditions or systemic abnormalities. No clinical signs or symptoms suggestive of syndromic involvement or abnormalities in other ectodermal‐derived organs were observed upon examination. Recent follow‐up examinations of both LBD1 and LBD2 individuals revealed stable occlusion and periodontal status, with no clinical signs of caries and good oral hygiene.

### 3.2 Alterations in Mesiodistal Crown Width

Mesiodistal crown diameters of most teeth in individuals with LBD1 and LBD2 were generally reduced compared to reference values. Lower incisors, however, showed mesiodistal diameters similar to the reference values. Notable exceptions within LBD1 included a significantly enlarged mandibular left central incisor (*Z*‐score = 2.64) and a significantly reduced maxillary left second molar (*Z*‐score = −2.26). In LBD2, the mandibular right lateral incisor also showed significant enlargement (*Z*‐score = 2.64) while the maxillary right lateral incisor was significantly reduced (*Z*‐score = −3.99) (Table 1).

**TABLE 1:** Mean mesiodistal widths (mm) and *Z*‐scores of LBD1 and LBD2 teeth compared to the reference data from 67 Thai males.

	Tooth number	Reference		LBD1		LBD2	
		Mean	SD	Mean	*Z*‐scores	Mean	*Z*‐scores
Maxilla	11	8.71	0.57	9.17	0.82	8.49	−0.38
	12	8.50	0.65	7.33	−1.80	5.92	**−3.99**
	13	8.23	0.48	8.17	−0.13	NA	NA
	14	7.64	0.42	7.68	0.11	7.21	−1.01
	15	7.06	0.53	7.08	0.04	NA	NA
	16	10.64	0.61	10.54	−0.16	10.42	−0.37
	17	10.29	0.76	9.57	−0.95	NA	NA
	21	8.67	0.58	8.57	−0.18	8.39	−0.48
	22	7.19	0.66	6.35	−1.27	6.78	−0.63
	23	8.13	0.49	8.18	0.08	7.54	−1.21
	24	7.60	0.49	7.38	−0.44	NA	NA
	25	7.12	0.53	7.47	0.65	NA	NA
	26	10.70	0.73	9.91	−1.09	10.08	−0.85
	27	10.39	0.67	8.88	**−2.26**	10.16	−0.34
Mandible	41	5.48	0.36	5.59	0.30	5.82	0.93
	42	6.09	0.46	6.34	0.56	7.29	**2.64**
	43	7.16	0.46	7.14	−0.04	7.43	0.57
	44	7.52	0.57	7.26	−0.46	7.75	0.41
	45	7.51	0.60	6.63	−1.49	7.58	0.11
	46	11.67	0.79	11.58	−0.12	12.47	1.00
	47	11.26	0.79	10.78	−0.61	12.51	1.58
	31	5.47	0.38	6.48	**2.64**	5.65	0.47
	32	6.10	0.41	NA	NA	6.64	1.30
	33	7.19	0.47	7.17	−0.05	7.38	0.39
	34	7.55	0.58	7.29	−0.45	7.40	−0.26
	35	7.60	0.57	7.28	−0.57	7.22	−0.67
	36	11.77	0.66	11.34	−0.64	12.36	0.89
	37	11.35	0.85	10.62	−0.87	NA	NA

*Note:* Tooth numbers are designated according to the FDI numbering system; Bolded values indicate significant differences from the normal value.

Abbreviations: LBD, lobodontia; NA, not available; SD, standard deviation.

### 3.3 Abnormal Morphology and Surface Features

**FIGURE 2:**
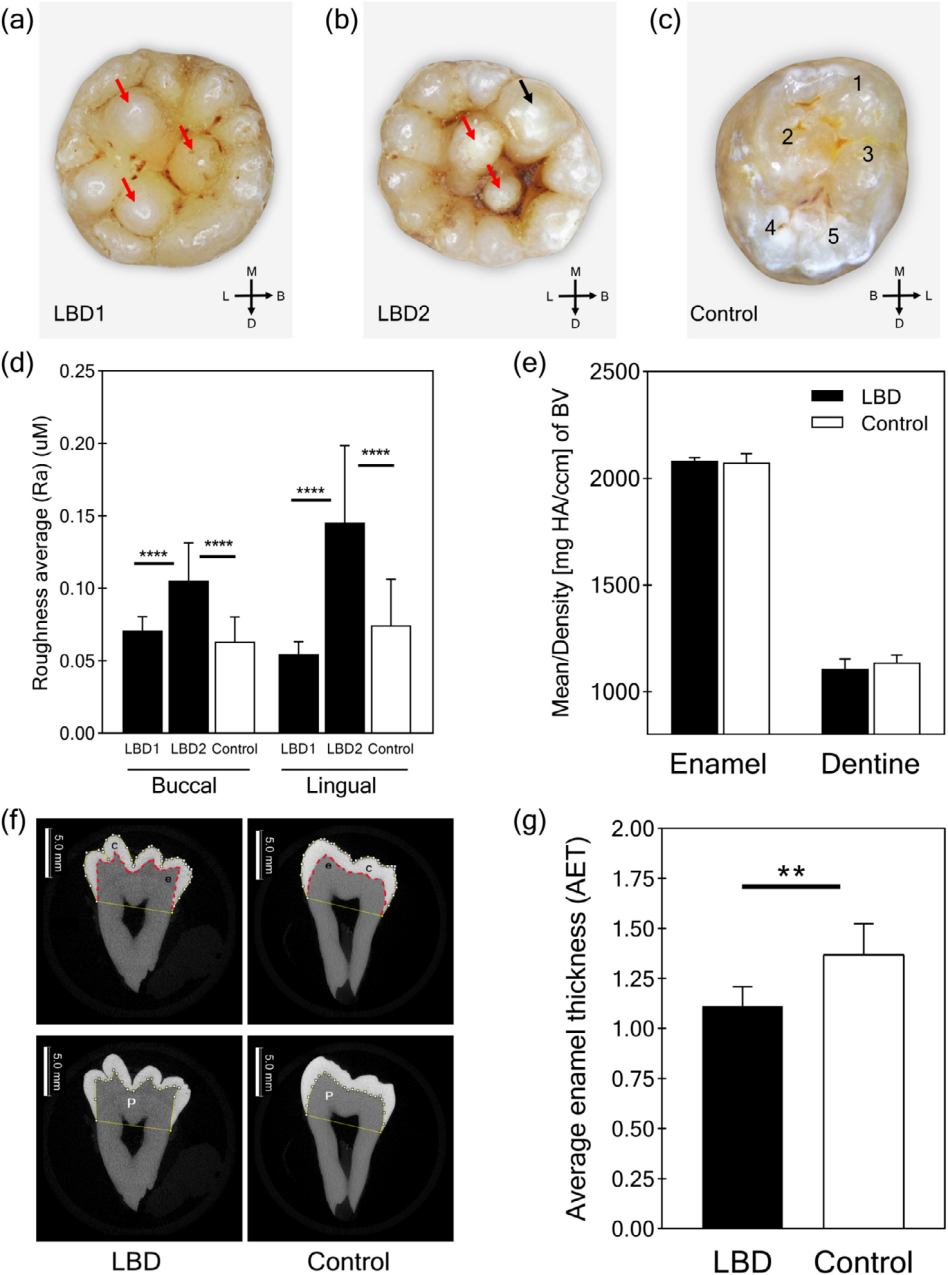
Tooth morphology, surface roughness, microcomputerized tomography scanning, and average enamel thickness. The permanent mandibular third molars obtained from LBD1 (a) and LBD2 (b) exhibited a “rosette‐like” multitubercular surface, characterized by multiple cusps, deep grooves, and pits, while the healthy control (c) displayed pyramidal‐shaped cusps and shallow grooves. M, Mesial; D, Distal; B, Buccal; L, Lingual. Red arrows indicate central supernumerary cusps. The black arrow indicates a tuberculated cusp on the labial lobe. Cusp numbers are addressed for control teeth. (d) Average surface roughness on buccal and lingual surfaces of LBD2 is significantly higher than control, while LBD1 is comparable to the controls (*n* = 5 teeth). (e) The mineral density of LBD teeth is not significantly different from controls (*n* = 6 teeth). (f) Micro‐CT sectional images of LBD and control teeth, along with parameters used for enamel thickness calculation, are demonstrated. C, tooth crown area; P, dentine under the enamel cap area; e, length of the enamel‐dentine junction (red‐dotted line). (g) The average enamel thickness values of LBD teeth are significantly lower than control teeth (*n* = 3 teeth). In all experiments, the data are presented in a bar graph as mean ± SD. **p value *≤* 0.01, ****p value ≤ 0.0001.

Lobodontia teeth (LBD1 and LBD2) exhibited marked abnormalities in crown morphology, characterized by a significant increase in cusp number, often exceeding 10 cusps. Occlusal surfaces displayed a distinctive multitubercular pattern with numerous peripheral and interstitial accessory cusps, resembling “mulberry molars” and resulting in pronounced pits and grooves (Figure 2a,b). Notably, LBD2 presented with a prominent tuberculated cusp on the buccomesial lobe (Figure 2b). In contrast, control teeth exhibited a typical morphology with fewer, less prominent cusps, shallower pits and grooves, pyramidal cusps, flattened buccal surfaces, and a small, pointed distal cusp (Figure 2c).

Color analysis further revealed differences between lobodontia and control teeth. LBD1 exhibited a slightly greenish‐blue hue, while LBD2 appeared darker with redder and yellower tones (Table S1). The color difference value (Δ*E*) between LBD1 and the control group was 8.54, and the Δ*E* between LBD2 and the control group was 15.32. Both Δ*E* values were above 8, which is regarded as distinctly perceivable by human vision (Yamanel et al. 2010).

Surface roughness analysis revealed significant differences between the groups. While the buccal and lingual surface roughness of LBD1 was comparable to that of control teeth, LBD2 exhibited significantly higher roughness on both surfaces (Figure 2d). This suggests variations in the enamel surface texture among lobodontia‐affected teeth. Micro‐CT analysis showed no significant differences in the mineral density of enamel and dentine between LBD and control teeth (Figure 2e and Table S2). However, in terms of enamel thickness, both LBD1 and LBD2 exhibited a significant reduction in average enamel thickness (AET) (1.112 mm) compared to control teeth (1.369 mm) (Figure 2f,g and Table S3). This finding points to a potential defect in enamel formation or maturation in lobodontia.

### 3.4 Compromised Mechanical Properties

**FIGURE 3:**
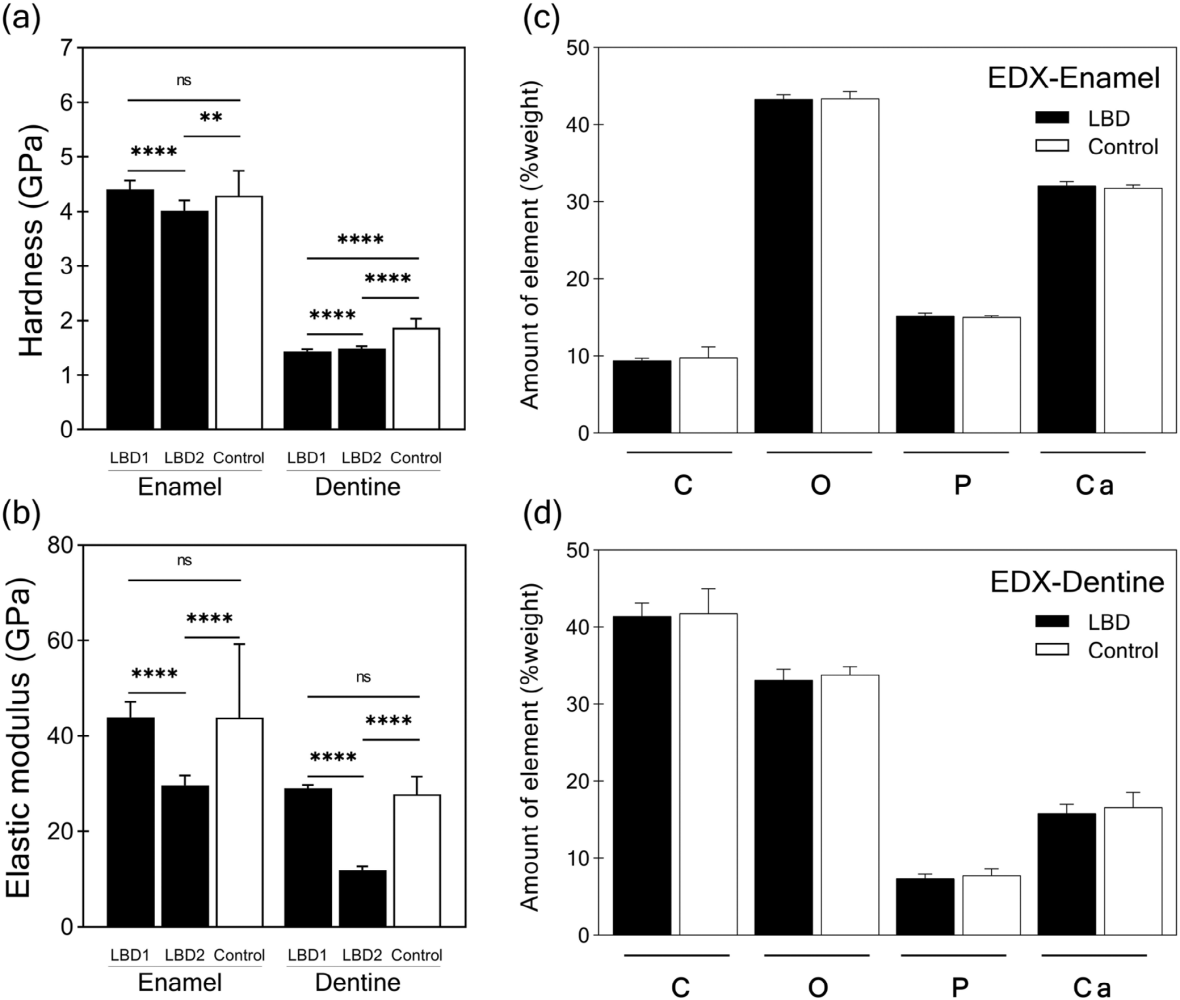
Measurement of nanohardness, elastic modulus, and mineral contents. (a) Nanohardness and (b) elastic modulus of LBD2 are significantly low compared with control teeth (*n* = 4 teeth). (c) The enamel and (d) dentine mineral contents are illustrated as percent weight values. C, carbon; Ca, calcium; O, oxygen; P, phosphorus. Data are presented as mean ± SD. **p value ≤ 0.011, ****p value ≤ 0.0001.

LBD2 exhibited significantly lower nanohardness and elastic modulus in both enamel and dentine compared to both control teeth and LBD1 (except for dentine hardness). LBD1, however, showed a significant reduction in dentine hardness compared to controls (Figure 3a,b). The elemental composition of enamel and dentine in LBD teeth, including carbon, oxygen, phosphate, and calcium content, was comparable to that of controls (Figure 3c,d). Furthermore, no significant differences were observed in the calcium/phosphate ratios between the groups (Figure S3). These findings were consistent across all regions examined, including the occlusal, middle, and cervical thirds of the tooth crown (Figure S4).

### 3.5 Ultrastructural Disintegration With Dentinoenamel Junction Gap

**FIGURE 4:**
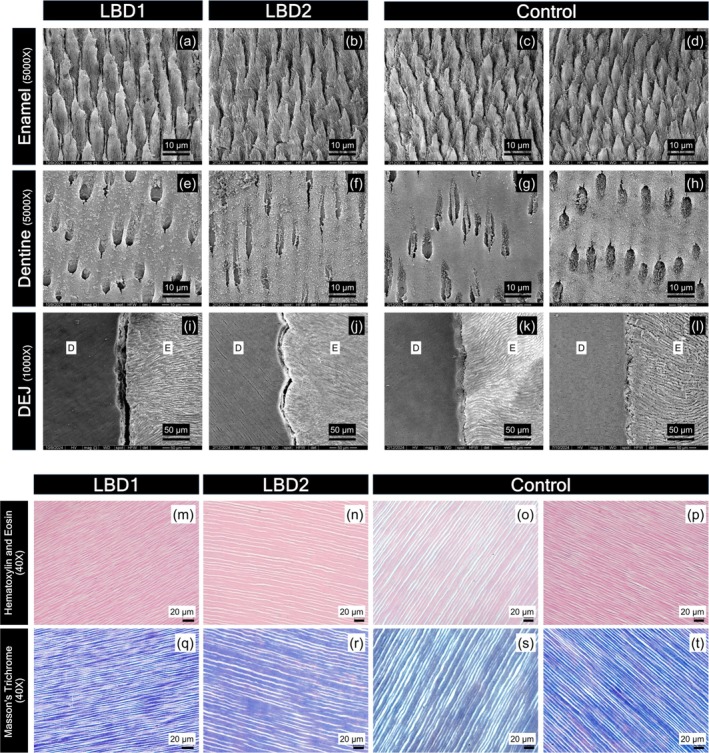
Ultrastructure of LBD teeth. SEM of LBD enamel displays regular arrangement and diameter of enamel prisms compared to the control (a–d). Dentine appearance of LBD teeth is comparable to control (e–h). Compared to the control, the dentinoenamel junction (DEJ) of LBD shows gap formation between dentine and enamel (i–l). Images of enamel and dentine were taken at 5000×, while the DEJ was imaged at 1000×. E, enamel; D, dentin. Histology analysis of LBD teeth. Hematoxylin and eosin (H&E) staining shows regularity of dentinal tubules in the dentine of LBD1 and LBD2 compared to controls (m–p). Masson's Trichrome staining shows consistent distribution of collagen in LBD1 and LBD2 dentine (q–t). H&E and Masson's Trichrome staining images were taken at 40×.

SEM revealed a generally normal ultrastructure in LBD teeth. Enamel prisms exhibited a regular arrangement comparable to controls (Figure 4a–d), and dentinal tubules with intertubular dentine appeared normal (Figure 4e–h). However, a striking observation was the presence of gap formations at the dentinoenamel junction (DEJ) in LBD teeth, while controls showed an organized scallop pattern of DEJ (Figure 4i–l). Histological analysis corroborated the normal appearance of dentinal tubules and collagen fiber organization in LBD dentine (Figure 4m–t).

## 4 Discussion

Lobodontia is characterized by a complex array of dental abnormalities. Previous research has documented various anomalies, including a “rosette‐like” multitubercular surface, supernumerary cusps, dens invaginatus in the molar crowns, and single pyramidal roots with a single root canal (Ather et al. 2013; Brook and Winder 1979; Gheorghe et al. 2023; Metgud et al. 2009; Nguyen et al. 1996; Skrinjaric et al. 2016). In line with previous findings, the molar crowns in LBD exhibited a multitubercular surface with multiple cusps, resulting in narrow, deep grooves and pits. Skrinjaric et al. (2016) reported reduced canine and premolar crown diameters in Croatian male individuals with lobodontia (Skrinjaric et al. 2016). Similarly, this study observed reduced crown width in most teeth of LBD1 and LBD2. While lower incisor dimensions were comparable to reference values, notable exceptions included significant enlargement of the mandibular left central incisor in LBD1 and the mandibular right lateral incisor in LBD2. This observed variability in tooth size within lobodontia may reflect genetic heterogeneity, potentially influenced by founder effects, unique allelic variants, or polygenic interactions. Further investigation incorporating comparative population studies, genetic analyses, detailed morphometric assessments, and consideration of environmental factors is needed to elucidate the etiological factors underlying tooth size variation in lobodontia.

The LBD2 tooth, from the older patient, exhibited more severe discoloration characterized by pronounced brown staining within the occlusal grooves and pits compared to both the control and LBD1 (younger patient). This was accompanied by greater surface roughness in LBD2. Furthermore, significant reductions in both enamel and dentine hardness and elasticity were observed in LBD2 compared to the control. These reductions were also observed in LBD2 compared to LBD1, particularly in enamel hardness and elastic modulus, and dentine elasticity. This suggests that age‐related changes in tooth structure may contribute to both the observed tooth discoloration and the deterioration of its mechanical properties.

Although less frequently documented, some reports have noted other anomalies in lobodontia patients such as taurodontism (Metgud et al. 2009) and dens invaginatus (Gheorghe et al. 2023; Metgud et al. 2009; Skrinjaric et al. 2016). In our study, detailed radiographic and cone‐beam CT analyses further expand this knowledge by identifying dens invaginatus type I in the maxillary incisors, canines, and premolars of the LBD1 patient, and type II, characterized by large and deep invaginations, in the maxillary incisors and premolars of LBD2. This more precise characterization contributes to the limited data on the co‐occurrence of dens invaginatus in lobodontia. This developmental anomaly arises from the infolding of the tooth crown surface prior to calcification. Previous research has indicated a reduction in the enamel area percentage observed via microradiographs in teeth exhibiting dens invaginatus (Crincoli et al. 2010). Additionally, prior studies have shown that teeth with dens invaginatus often demonstrate other structural anomalies in dental hard tissues, such as hypomineralized enamel and possess an increased risk of pulpal infection (Beynon 1982; Piattelli and Tris 1993). These anomalies also include a broader enamel prism diameter and a reduced number and diameter of dentinal tubules (Crincoli et al. 2010). Taken together, our findings, in conjunction with previous research, underscore the significant differences in enamel thickness and structural integrity between lobodontia‐affected teeth and normal control teeth, providing further insight into the complexities of dental anomalies in this condition.

The dentinoenamel junction serves as a crucial interface, preventing crack propagation from enamel into dentine due to the elastic modulus mismatch between these tissues (Bechtle et al. 2010). While lobodontia‐affected teeth displayed unremarkable enamel rods and dentinal tubules compared to controls, they exhibited a gap at the junction. This gap may contribute to a compromised elastic modulus and reduced nanohardness, potentially weakening the structural integrity of the tooth. These findings suggest that lobodontia may impact developmental processes at the DEJ, potentially affecting the formation and interaction of these tissues at that critical interface. The observed gaps at the DEJ may result from disruptions in the signaling pathways that regulate amelogenesis and dentinogenesis, as well as their coordinated integration during tooth development. *CACNA1S*, which encodes the α1S subunit of the L‐type voltage‐gated calcium channel, is a crucial regulator of intracellular calcium homeostasis (Schartner et al. 2017). Although the precise role of *CACNA1S* in odontogenesis remains to be fully elucidated, calcium signaling is vital for both enamel mineralization by ameloblasts and the differentiation and function of odontoblasts, which are responsible for dentine formation. It is hypothesized that dysfunction of *CACNA1S* could disrupt calcium dynamics within these cell populations, potentially compromising the formation and structural integrity of the DEJ. Further research, including both in vitro and in vivo models, is needed to investigate the specific contribution of *CACNA1S* to DEJ morphogenesis and to clarify the molecular mechanisms underlying the observed DEJ defects.

Tooth discoloration was observed in the lobodontia specimens, consistent with a prior report (Ather et al. 2013). This discoloration may result from structural changes in the thickness or composition of dental hard tissues. The compromised integrity of enamel and dentine could also contribute to these color changes. These deviations in color are likely attributable to alterations in enamel translucency or thickness, which may affect light scattering and, consequently, the overall appearance of the tooth.

Apart from the typical dental features of lobodontia, patients may also exhibit malocclusion, missing or impacted teeth, and periodontal issues such as alveolar bone loss. The presence of protruding cusps can contribute to malocclusion and abnormal occlusal forces, potentially leading to alveolar bone loss. Previous studies have reported endodontic treatment in several cases, with patient ages ranging from 22 to 52 years (Ather et al. 2013; Gheorghe et al. 2023; Metgud et al. 2009). Given the increased susceptibility of LBD patients to dental caries and structural fractures due to compromised enamel hardness and DEJ disintegration, specific clinical management strategies are essential to preserve tooth integrity and prevent complications. In addition to the use of remineralizing agents such as fluoride and calcium phosphate compounds to enhance enamel resistance, individualized preventive approaches should be prioritized. Gentle brushing techniques using ultra‐soft toothbrushes and non‐abrasive toothpaste may help reduce mechanical stress on weakened dental tissues. Orthodontic evaluation plays a role in the correction of traumatic occlusion, which may lead to progressive structural deterioration. Occlusal adjustments or protective appliances may also be indicated in cases of bruxism or malocclusion. Preventive sealants should be considered not only for occlusal surfaces but also for palatal or lingual pits, particularly in maxillary incisors affected by dens invaginatus, to reduce the risk of bacterial ingress and subsequent endodontic involvement. More frequent professional cleanings, fluoride applications, and dietary counseling to limit acidic exposures are also recommended. A multidisciplinary approach involving general dentists, pediatric dentists, operative dentists, and orthodontists is essential for delivering comprehensive, patient‐centered care. Future research should further evaluate the long‐term effectiveness of these targeted interventions in improving oral health outcomes in this vulnerable population.

A limitation of this study is the small sample size of extracted lobodontia teeth available for detailed phenotyping, which is inherent to the rarity of this condition, estimated at 1 in 300,000 (Skrinjaric et al. 2016). This limited sample may impact the generalizability of the findings. To assess the adequacy of our samples, we performed a G*Power analysis, which demonstrated that measurements of mineral density, hardness, and elastic modulus achieved sufficient statistical power (greater than 0.8). However, other experiments, including tooth color, surface roughness, enamel thickness, and mineral composition, did not reach this threshold. Given the paucity of affected teeth, increasing the sample size is currently not feasible. The findings from these rare cases provide initial yet important preliminary insights into the dental phenotype of lobodontia.

Collectively, our findings expand the phenotypic and structural spectrum of lobodontia and offer insights into its biomechanical implications. By clarifying both morphological and functional features, this study enhances understanding and informs improved clinical management of this rare dental anomaly.

Future research should prioritize collaborative efforts to increase the sample size for a more comprehensive analysis with improved statistical robustness, comparing developmental similarities and differences in dental abnormalities during early developmental stages, and investigating the causative factors and molecular pathomechanisms of lobodontia to obtain a more comprehensive understanding of its characteristics, developmental progression, and clinical implications for dental health.

In summary, this study underscores the unique dental morphology associated with lobodontia, demonstrating that lobodontia‐affected teeth exhibit significant structural and mechanical deficiencies. These findings highlight the need for further research to elucidate the underlying mechanisms responsible for these dental anomalies and to explore potential preventive and therapeutic strategies.

## Author Contributions

**Thanakorn Theerapanon:** data curation, investigation, formal analysis, writing – original draft. **Narin Intarak:** conceptualization, data curation, formal analysis, investigation, writing – original draft, writing – review and editing. **Sasiprapa Prommanee:** formal analysis, investigation, writing – review and editing. **Sunisa Somkana:** formal analysis, investigation. **Sirinya Kulvitit:** formal analysis, investigation. **Anucharte Srijunbarl:** formal analysis, investigation, writing – review and editing. **Junji Tagami:** formal analysis, investigation, writing – review and editing. **Thantrira Porntaveetus:** conceptualization, data curation, supervision, validation, resources, writing – review and editing, funding acquisition.

## Ethics Statement

The study was approved by the Research Ethics Committee of the Faculty of Dentistry, Chulalongkorn University, Bangkok, Thailand (No. 137/2023, HREC‐DCU2023‐117), and in accordance with the Declaration of Helsinki (version 2002) and the additional requirements.

## Consent

Informed consent was obtained from all participants involved in this study, encompassing approval for both the publication of data and photographs.

## Conflicts of Interest

The authors declare no conflicts of interest.

## Supporting information


**Figure S1.** Dental models of lobodontia patients. LBD1 (a), LBD2 (b), and mesiodistal width measurements using sliding calipers (c).
**Figure S2.** Dental phenotypes of LBD1’s sister and mother.
**Figure S3.** Calcium/phosphate ratio of enamel and dentine of LBD teeth compared to controls.
**Figure S4.** Elemental composition and calcium/phosphate ratio in tooth crowns.
**Table S1.** Tooth color measurement.
**Table S2.** Dental mineral density measured by micro computerized tomography.
**Table S3.** Enamel thickness measurement.

## Data Availability

The data that support the findings of this study are available from the corresponding author upon reasonable request.
