# Supplementary material for: Lobodontia‐Affected Teeth Exhibit Compromised Integrity and Enamel Defects: A Deep Phenotyping Study
Source: Oral Dis. 2025 May 25;31(11):3115–25. doi: 10.1111/odi.15390 (PMC12803554; doi:10.1111/odi.15390)
Supplement: Supplementary file 1 — Figure S1. Dental models of lobodontia patients. LBD1 (a), LBD2 (b), and mesiodistal width measurements using sliding calipers (c). Figure S2. Dental phenotypes of LBD1’s sister and mother. Figure S3. Calcium/phosphate ratio of enamel and dentine of LBD teeth compared to controls. Figure S4. Elemental composition and calcium/phosphate ratio in tooth crowns. Table S1. Tooth color measurement. Table S2. Dental mineral density measured by micro computerized tomography. Table S3. Enamel thickness measurement. [file ODI-31-3115-s001.docx]

**Supplementary Data**

**Lobodontia-Affected Teeth Exhibit Compromised Integrity and Enamel Defects: A Deep Phenotyping Study**

Thanakorn Theerapanon^1,2^, Narin Intarak^2^, Sasiprapa Prommanee^2,3^, Sunisa Somkana^2^, Sirinya Kulvitit^2,4^, Anucharte Srijunbarl^5^, Junji Tagami^6,7^, Thantrira Porntaveetus^2,8*^

^1^Interdisciplinary Program of Biomedical Sciences, Graduate School, Chulalongkorn University, Bangkok, 10330, Thailand

^2^Center of Excellence in Precision Medicine and Digital Health, Center of Excellence in Genomics and Precision Dentistry, Geriatric Dentistry and Special Patients Care International Program, Department of Physiology, Faculty of Dentistry, Chulalongkorn University, Bangkok, 10330, Thailand

^3^Clinical Research Center, Faculty of Dentistry, Chulalongkorn University, Bangkok, 10330, Thailand

^4^Department of Operative Dentistry, Faculty of Dentistry, Chulalongkorn University, Bangkok, 10330, Thailand

^5^Dental Materials R&D Center, Faculty of Dentistry, Chulalongkorn University, Bangkok, 10330, Thailand

^6^Faculty of Dentistry, Chulalongkorn University, Bangkok, 10330, Thailand

^7^Department of Cariology and Operative Dentistry, Graduate School of Medical and Dental Sciences, Institute of Science Tokyo, Tokyo, 113-8549, Japan.

^8^Clinic of General-, Special Care and Geriatric Dentistry, Center for Dental Medicine, University of Zurich, Zurich, 8032, Switzerland


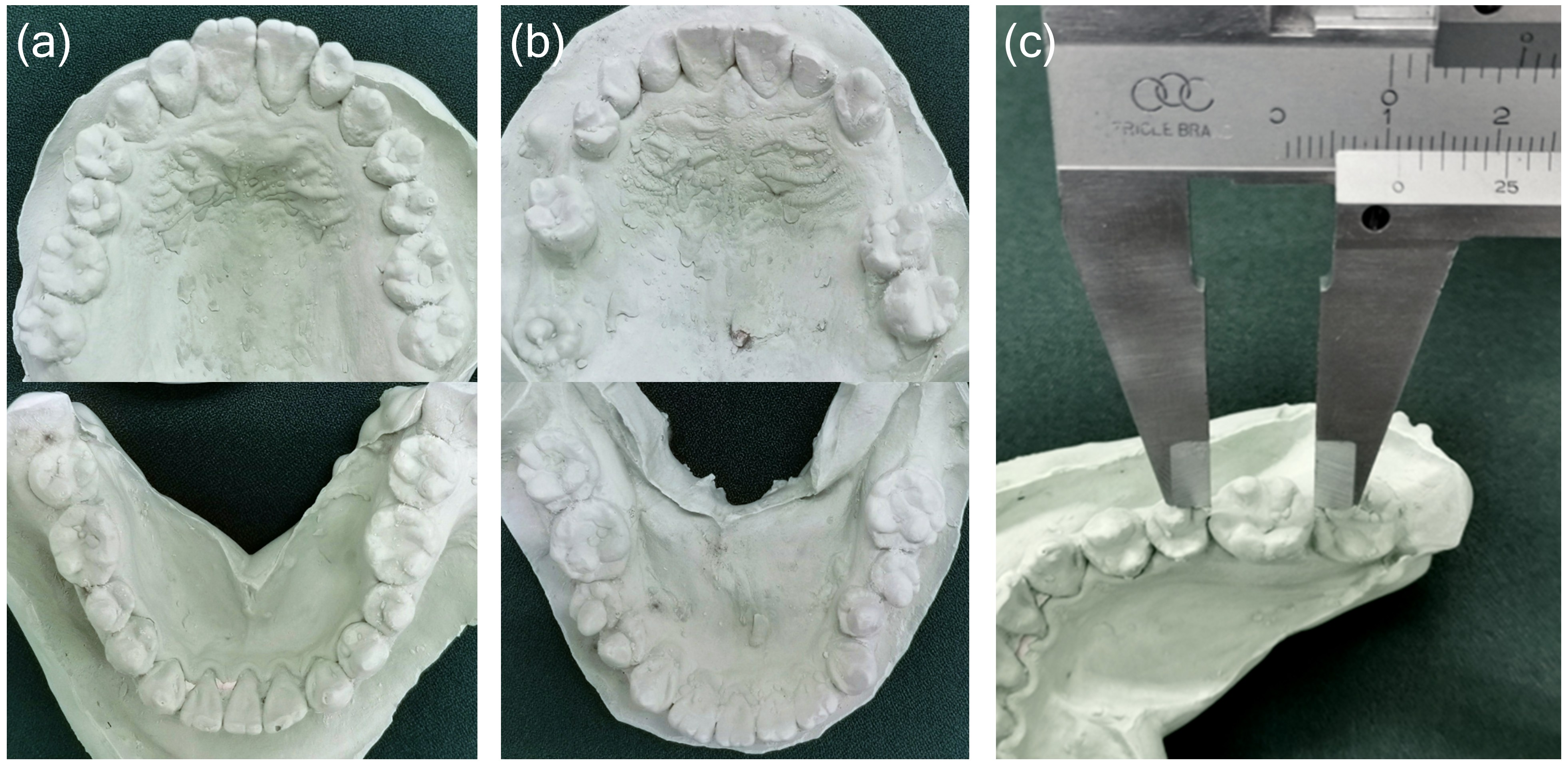


**Figure S1** **Dental models of lobodontia patients.** Dental models of LBD1 (a), LBD2 (b), and mesiodistal width measurements using sliding calipers (c).

**
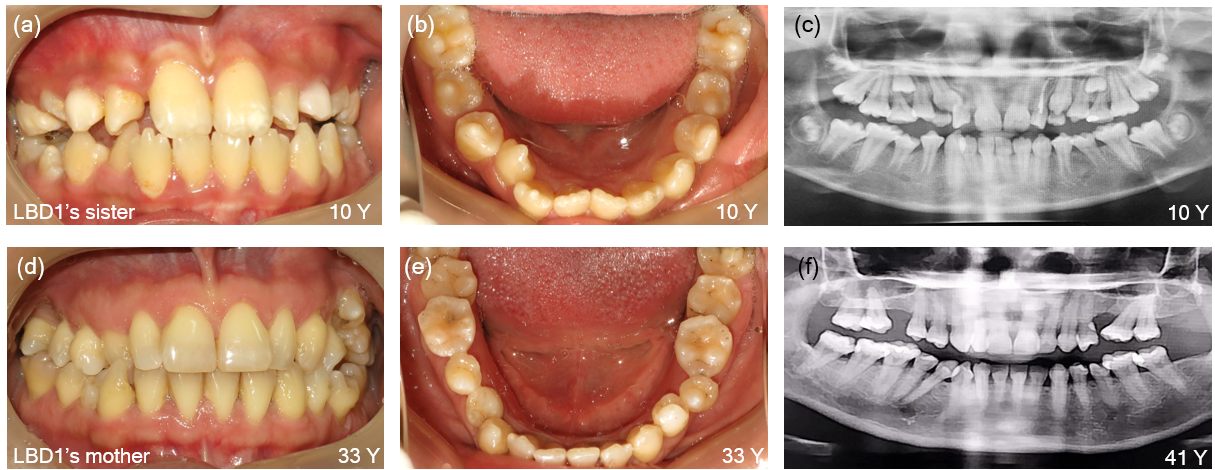
**

**Figure S2 Dental phenotypes of LBD1's sister and mother.** (a-c) LBD1's sister exhibited lobodontia features, including tritubercular incisors, single-cusped premolars, multitubercular molars with single roots, and malocclusion. The permanent maxillary left lateral incisor had undergone endodontic treatment. (d-f) LBD1's mother also exhibited typical lobodontia features, including attrition of her incisors and malocclusion. The maxillary right first molar and maxillary left second premolar were previously extracted.


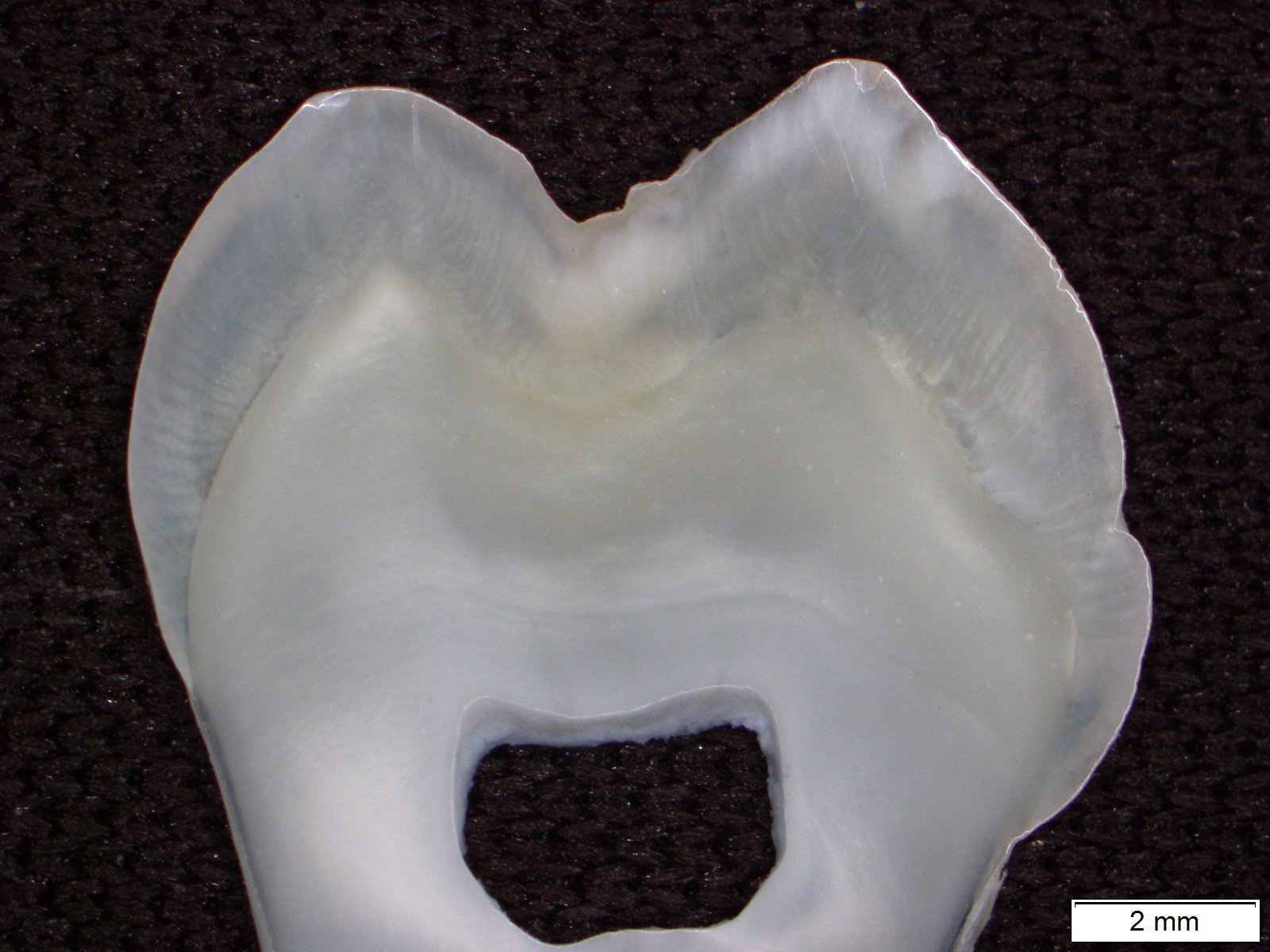

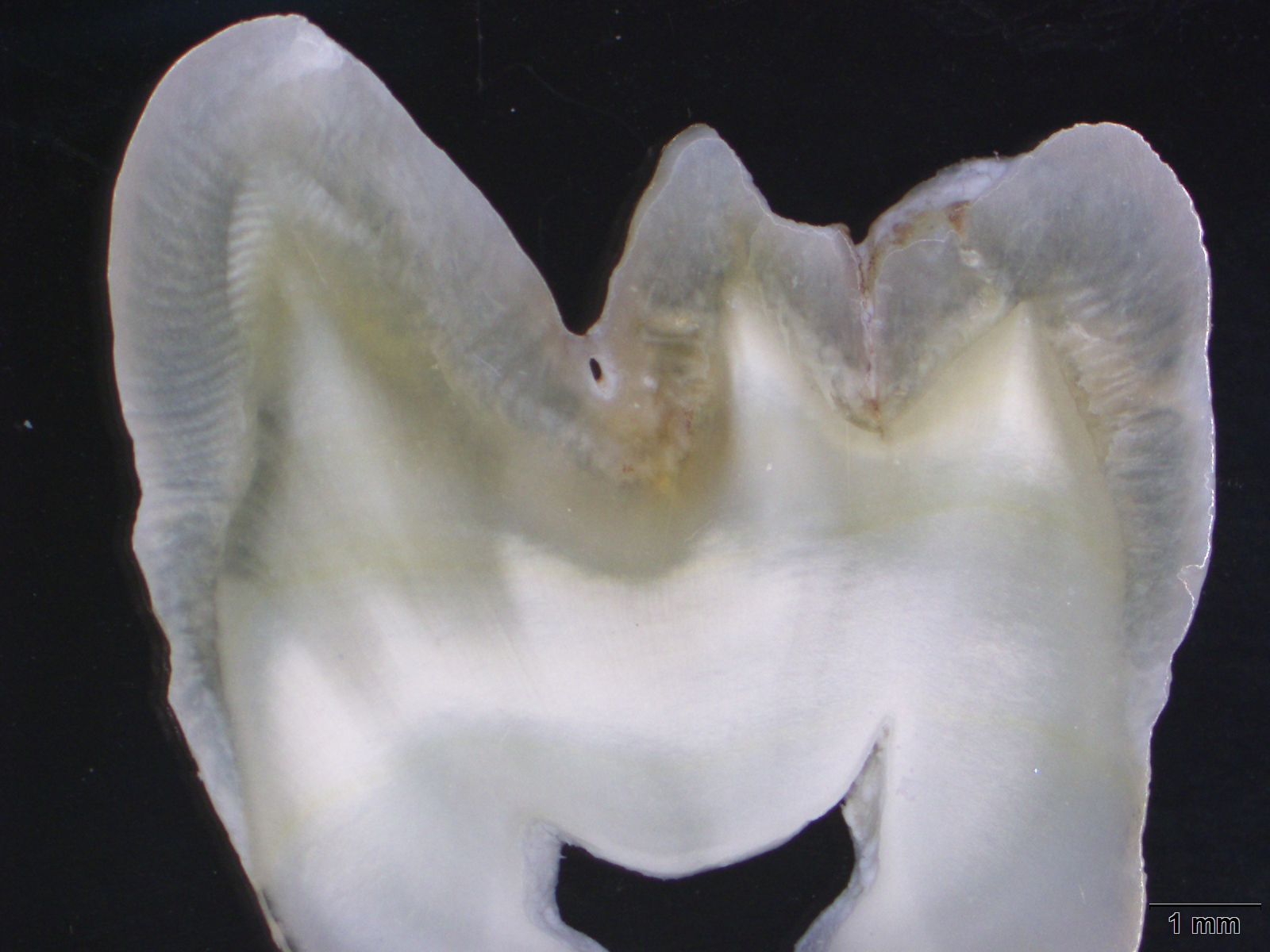


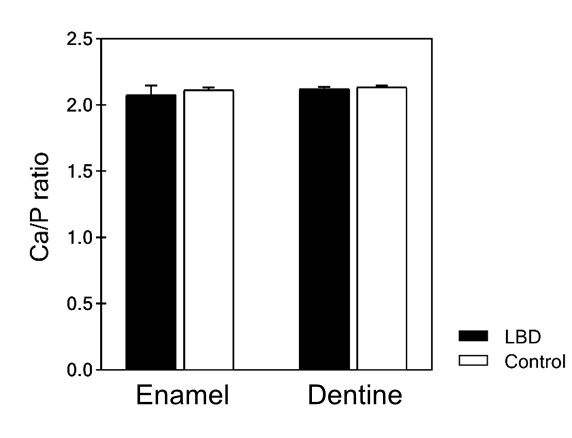


**Figure S3** **Calcium/phosphate ratio of enamel and dentine of LBD teeth compared to controls.**


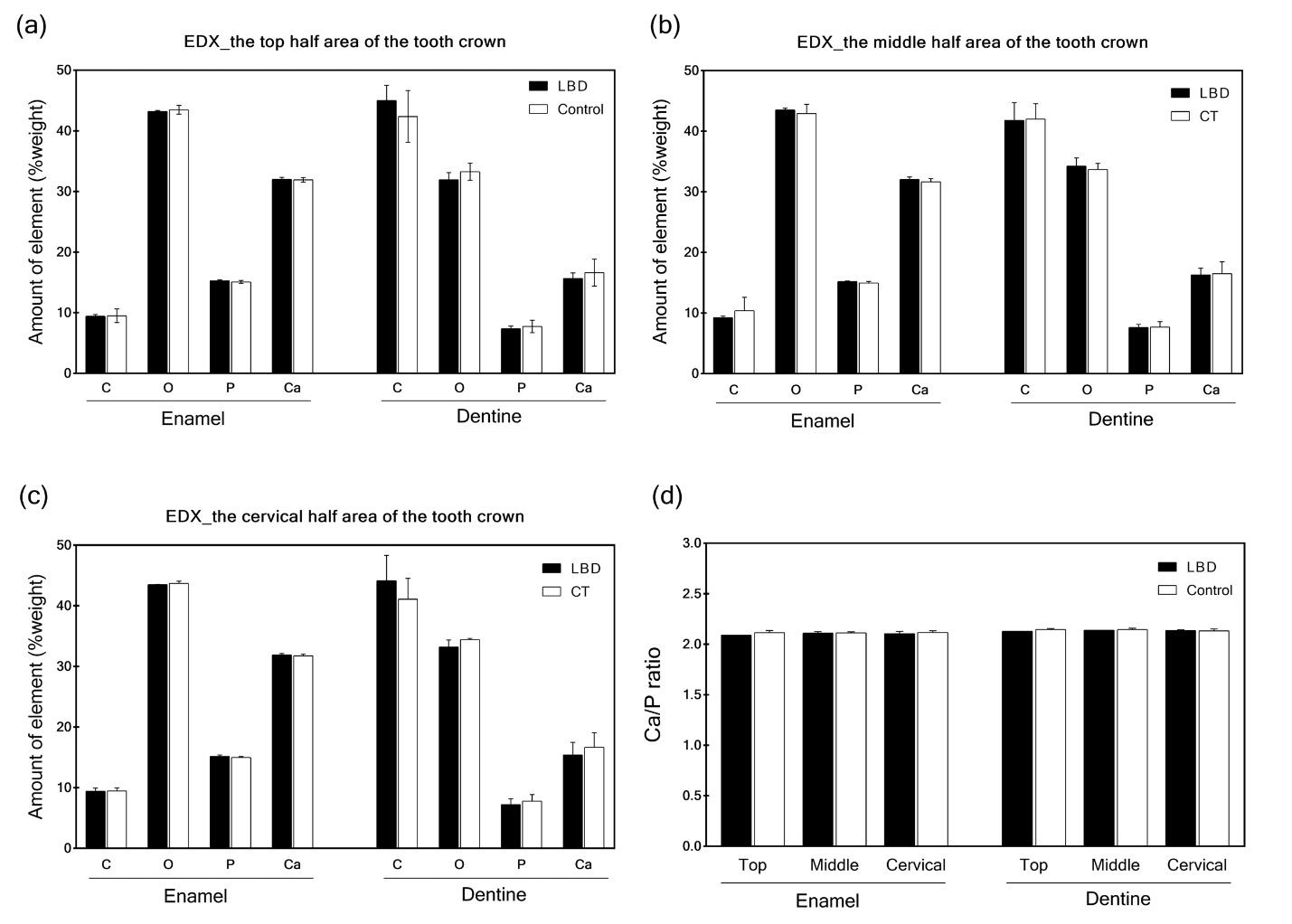


**Figure S4. Elemental composition and calcium/phosphate ratio in tooth crowns.** Elemental analysis of enamel and dentine in (a) the coronal third, (b) the middle third, and (c) the cervical third of the tooth crown. (d) Comparison of calcium/phosphate ratios in enamel and dentine between LBD teeth and controls. Mineral content is expressed as weight percentage. C, carbon; O, oxygen; P, phosphorus; Ca, calcium. Data are presented as mean ± standard deviation (SD).

**Table S1.** **Tooth colour measurement.**

LBD, Lobodontia; CT, Control

| Samples | L* (lightness) | a* (green-red) | b* (blue-yellow) | ΔE* (LBD vs control) |
| --- | --- | --- | --- | --- |
| LBD-1 | 89.85 | -2.9 | 24.25 | - |
| ΔE average |  |  |  | 8.54 |
| CT1 | 92.40 | 0.95 | 31.80 | 8.85 |
| CT2 | 81.05 | -1.90 | 26.90 | 9.24 |
| CT3 | 87.50 | -2.80 | 26.20 | 3.06 |
| CT4 | 80.95 | -1.35 | 33.05 | 12.61 |
| CT5 | 85.25 | -1.45 | 31.80 | 8.96 |
| LBD-2 | 76.80 | 1.00 | 41.70 | - |
| ΔE average |  |  |  | 15.32 |
| CT1 | 92.40 | 0.95 | 31.80 | 18.52 |
| CT2 | 81.05 | -1.90 | 26.90 | 15.67 |
| CT3 | 87.50 | -2.80 | 26.20 | 19.23 |
| CT4 | 80.95 | -1.35 | 33.05 | 9.89 |
| CT5 | 85.25 | -1.45 | 31.80 | 13.27 |

**Table S2.** **Dental mineral density measured by micro-computerised tomography**

| **Sample** | **Enamel** | **Dentine** |
| --- | --- | --- |
| **LBD1 average** | **2088.772** | **1070.368** |
| LBD1 | 2085.394 | 1058.666 |
|  | 2103.851 | 1083.185 |
|  | 2077.070 | 1069.252 |
| **LBD2 average** | **2075.286** | **1142.366** |
| LBD2 | 2072.823 | 1183.276 |
|  | 2089.822 | 1127.829 |
|  | 2063.211 | 1115.992 |
| **LBD average** | **2082.029** | **1106.367** |
| **CT average** | **2074.076** | **1136.263** |
| CT1 | 2033.911 | 1142.009 |
|  | 2132.827 | 1138.059 |
|  | 2040.248 | 1141.458 |
| CT2 | 2105.549 | 1098.566 |
|  | 2081.945 | 1116.201 |
|  | 2057.882 | 1074.320 |
| CT3 | 2112.339 | 1107.448 |
|  | 2070.153 | 1117.149 |
|  | 2138.772 | 1154.885 |
| CT4 | 2085.283 | 1122.044 |
|  | 2010.079 | 1122.756 |
|  | 2093.649 | 1144.116 |
| CT5 | 2076.650 | 1225.105 |
|  | 2026.989 | 1167.612 |
|  | 2048.794 | 1174.643 |
| CT6 | 2118.747 | 1103.176 |
|  | 2001.001 | 1175.889 |
|  | 2098.544 | 1127.295 |
|  |  |  |

LBD, Lobodontia; CT, Control

**Table S3**. **Enamel thickness measurement.**

| **Sample** | **C** | **P** | **c** | **e** | **c/e** |
| --- | --- | --- | --- | --- | --- |
| LBD1.1 | 57.7 | 36.1 | 21.6 | 20.67 | 1.045 |
| LBD1.2 | 55.917 | 34.477 | 21.44 | 20.315 | 1.055 |
| LBD1.3 | 56.018 | 34.284 | 21.734 | 21.733 | 1.000 |
| LBD2.1 | 68.914 | 41.042 | 27.872 | 22.18 | 1.257 |
| LBD2.2 | 67.087 | 41.34 | 25.747 | 22.532 | 1.143 |
| LBD2.3 | 62.299 | 38.85 | 23.449 | 19.961 | 1.175 |
| **LBD average** |  | | | | 1.112 |
| **CT average** |  | | | | 1.369 |
| CT1.1 | 64.104 | 39.937 | 24.167 | 17.682 | 1.367 |
| CT1.2 | 62.771 | 42.015 | 20.756 | 18.989 | 1.093 |
| CT1.3 | 69.114 | 45.718 | 23.396 | 19.195 | 1.219 |
| CT2.1 | 64.467 | 38.239 | 26.228 | 17.771 | 1.476 |
| CT2.2 | 64.22 | 36.889 | 27.331 | 17.533 | 1.559 |
| CT2.3 | 68.315 | 40.298 | 28.017 | 17.825 | 1.572 |
| CT3.1 | 51.772 | 30.032 | 21.74 | 15.535 | 1.399 |
| CT3.2 | 53.065 | 31.971 | 21.094 | 15.975 | 1.320 |
| CT3.3 | 50.17 | 29.957 | 20.213 | 15.339 | 1.318 |

C, tooth crown area; P, dentine under the enamel cap area; c, enamel surface area is derived by subtracting C from P; e, enamel-dentine junction length; c/e, The average thickness of the enamel (AET)
